# Two-Level Theory of Second-Order Nonlinear X‑ray Response beyond the Electric-Dipole Approximation

**DOI:** 10.1021/acs.jpca.5c07630

**Published:** 2026-01-13

**Authors:** Abhinay V. Mohan, Carles Serrat

**Affiliations:** † Department of Physics, 16767 Universitat Politècnica de Catalunya, Ronda de Sant Nebridi 22, Terrassa 08222, Spain; ‡ Aix−Marseille Université, Faculté de Physique, Marseille 13013, France

## Abstract

We develop a two-level theory of the second-order nonlinear X-ray response beyond the electric-dipole approximation, deriving the leading quadrupolar correction originating from interference with the dipolar pathway at the amplitude level. A compact scaling law links the correction to weighted linear-response oscillator strengths, allowing parameter-free estimates across different core edges within the limits of the two-level description. For difference frequency resonant with the core transition, within the two-level description adopted here, the frequency dependence of the observable beyond-dipole correction is set by the electric dipole–quadrupole pathway through field gradients and is controlled by the dimensionless factor (ω_1_ + ω_2_)^2^/Ω_0_^2^ = (2*r* – 1)^2^ with *r* = ω_1_/Ω_0_, while the underlying dipole–quadrupole interference occurs at the amplitude level and cancels in the isotropically averaged intensity, leaving a small positive quadratic correction whose magnitude is estimated from an isotropic linear-response oscillator-strength ratio. In particular, for a two-color scheme with ω_1_ = 4Ω_0_ and ω_2_ = 3Ω_0_, the quadrupolar contribution modifies the difference-frequency intensity by about 1.3% at the O *K* edge of CO and 5.5% at the S *K* edge of cysteine, consistent with the (2*r* – 1)^2^ dependence and the growth of *f* ^(2)^ with core energy. In liquids and gases, where the emitted difference-frequency field is strongly reabsorbed at the core edge, the relevant observable is the per-molecule nonlinear conversion efficiency obtained from orientationally averaged single-molecule emission. After isotropic averaging with linear polarizations, the dipole–quadrupole interference term vanishes by symmetry at the intensity level, so the observable correction arises solely from the surviving quadratic beyond-dipole contribution and follows a unified scaling. The same two-level structure carries over to sum-frequency generation with a reduced nondipole prefactor. The model targets molecules in the gas phase or solution and does not address oriented or crystalline systems. These results provide a practical rule for estimating beyond-dipole effects in second-order X-ray mixing and clarify when a dipole-only analysis becomes inadequate.

## Graphical abstract

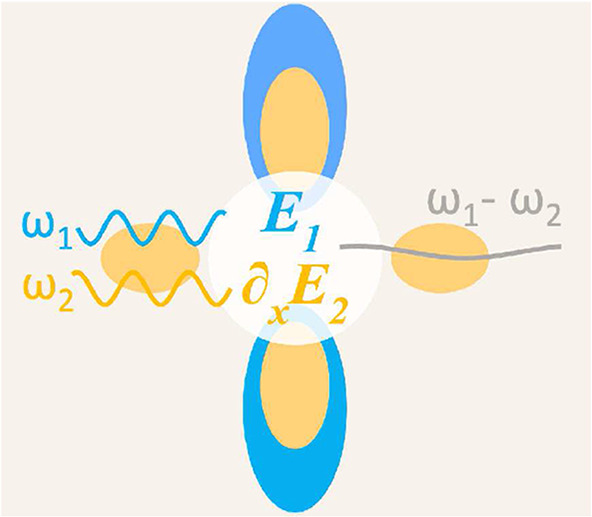

## Introduction

Nonlinear X-ray spectroscopy has emerged as a powerful route to probe ultrafast electronic dynamics at the atomic scale, enabled by intense coherent X-ray sources such as free-electron lasers (XFELs).^1–3^ Among second-order processes, resonant X-ray difference-frequency generation (re-XDFG) is particularly appealing^4,5^ because two-color X-ray pulses generate radiation at their frequency difference tuned to a core absorption edge, enabling element- and site-selective excitation with large penetration depth. Numerical simulations have demonstrated feasibility in small molecules,^4^ and subsequent work has highlighted sensitivity to symmetry and geometry.^5^

In liquids and gases, the difference-frequency field generated at a core edge is strongly reabsorbed in the bulk. The physically relevant quantity is therefore the per-molecule nonlinear conversion efficiency extracted from orientationally averaged single-molecule emission rather than a macroscopic coherent χ^(2)^ field. This viewpoint aligns the re-XDFG observable with indirect readouts such as UV transients and frames percent-level nondipole corrections as changes in the microscopic conversion efficiency. Most theoretical treatments of X-ray wave mixing adopt the electric-dipole approximation, which is valid when the external field is essentially uniform across the molecular charge distribution, that is, when the dimensionless parameter *kr_c_* ≪ 1 for a characteristic size *r_c_*. In that limit, the multipole expansion is dominated by the dipole term, and higher orders such as electric quadrupole and magnetic dipole are suppressed by powers of *kr_c_*. At optical wavelengths (λ ∼ 500 nm) with *r_c_* ∼ 1 Å, one has *kr_c_* ∼ 10^–3^. At X-ray wavelengths (λ ∼ 1 Å), one finds ${kr}_{c}=O\left(1\right)$ , so beyond-dipole contributions can appreciably modify nonlinear signals. In practice, electric quadrupole and magnetic dipole couplings can become relevant, and accurate modeling of oscillator strengths calls for explicit beyond-dipole treatments in a gauge-consistent multipolar framework^6–9^ based on the Power–Zienau–Woolley formalism.^10^ In the optical regime, beyond-dipole terms are not strictly zero but are negligible, so the electric dipole approximation and its selection rules provide an accurate description.

Within a two-level description, understood as an effective reduction of the near-edge manifold to a single dominant resonant channel |0⟩ → |*b*⟩, the beyond-dipole correction admits a unified isotropic scaling that links the observable percent change to a ratio of linear oscillator strengths. Writing *r* ≡ ω_1_/Ω_0_ with ω_2_ = ω_1_ – Ω_0_ and defining *R* ≡ |*f* ^(2)^|/*f* ^(0)^ from isotropic linear-response data, the orientationally averaged intensity change follows

$$\delta I\left(r\right)=\frac{10}{27}{\left(2r-1\right)}^{2}R$$

which is independent of the common Lorentzian line shape under exact resonance within the two-level model. This scaling identifies regimes where nondipole effects are no longer negligible and provides a parameter-free estimate once *R* is known from standard linear calculations.

Here, we develop a compact theoretical framework for second-order X-ray responses that incorporates beyond-dipole effects. Using a two-level model, we derive analytical expressions for quadrupolar contributions and their interference with dipolar pathways. Although minimal in structure, the framework captures the essential physics of core-edge resonances and connects directly to beyond-dipole oscillator strengths from *ab initio* linear-response calculations. As an illustrative case, we analyze resonant X-ray difference frequency at the oxygen *K* edge in CO and the sulfur *K* edge in cysteine, and we quantify quadrupolar corrections to the nonlinear response. More generally, the formalism applies to any second-order X-ray process and provides a transparent basis for interpreting nonlinear spectroscopy beyond the dipole approximation.

Throughout this article, we compute the single-molecule re-XDFG emission, and for isotropic ensembles, we average the emitted intensity over molecular orientations, ⟨|*A* ^(2)^|^2^⟩_orient_, which is the appropriate observable for an incoherent collection of molecules. In the electric-dipole approximation, this procedure reproduces the known selection rule, nonzero for noncentrosymmetric species such as CO and zero for centrosymmetric species such as CO_2_. In the hard X-ray regime, the beyond-dipole *k*-linear electric-dipole–electric-quadrupole term yields a finite single-molecule emission even for centrosymmetric molecules, and this is the correction quantified below.

The model targets molecules in the gas phase or solution and does not address oriented or crystalline systems, where tensorial χ^(2)^ responses and local-field effects dominate. For isotropic samples measured in intensity with linear polarizations, the present framework recovers the dipolar selection rule in the optical limit while retaining the finite *k*-linear dipole–quadrupole contribution that becomes relevant at hard X-rays.

## Theoretical Framework

The matter–radiation interaction can be expressed through the multipole expansion^11,12^ developed here in the semi-classical Power–Zienau–Woolley multipolar gauge for a monochromatic, linearly polarized plane wave propagating along *x*. Truncating at electric–dipole and electric–quadrupole order yields the effective light–matter Hamiltonian

1 $${Ĥ}_{\mathrm{int}}\left(t\right)=-\mathrm{\mu ̂}E\left(x,t\right)-\frac{1}{6}\mathrm{Q̂}\partial_{x}E\left(x,t\right)+...$$

where μ̂ is the electric–dipole operator and *Q̂* is the traceless electric–quadrupole operator. Magnetic–dipole terms are of the same formal order *kr_c_*. For isotropic samples measured in intensity with parallel linear polarizations, magnetic–dipole couplings contribute to the molecular response at the amplitude level but do not generate an interference term in the orientationally averaged intensity and thus do not introduce an independent frequency scaling (see Supporting Information for the general derivation including magnetic-dipole terms). Within the two-level approach, they can only renormalize the overall beyond-dipole magnitude, so the compact scaling below is quantitatively accurate when the magnetic contribution is subdominant. The multipolar Power–Zienau–Woolley expansion used here is equivalent to the minimal-coupling Hamiltonian up to the same order in *kr_c_*. We adopt it because it separates dipolar, quadrupolar, and magnetic pathways explicitly, providing direct physical insight and a simple scaling law for their relative weights. The minimal-coupling form indeed contains all multipoles but does not isolate their contributions in a transparent way, which makes the present formulation more practical for quantifying beyond-dipole effects at hard X-ray energies.

To analyze the X-ray difference-frequency component within the two-level setting, we model two monochromatic plane-wave drivers,

2 $$E_{j}\left(x,t\right)=E_{j}e^{i\left(k_{j}x-\omega_{j}t\right)}+\mathrm{c.c.},,k_{j}=\omega_{j}/c\;\left(j=1,2\right)$$

retaining the spatial phase so that the quadrupolar coupling involves ∂_*x*_ *E*_*j*_ = i*k*_*j*_ *E*_*j*_. After taking the derivative, we evaluate the local response at the molecular center *x* = 0,

3 $$E\left(t\right)=E_{1}e^{-i\omega_{1}t}+E_{2}e^{-i\omega_{2}t}+\mathrm{c.c.},,\omega_{3}=\omega_{1}-\omega_{2}$$

which selects the difference-frequency output resonant with the core transition.

Within standard nonlinear response theory,^13,14^ the induced polarization is expanded as a power series in the applied field. The quadratic susceptibility χ^(2)^ governs parametric generation at ω_3_ = ω_1_ – ω_2_ in the X-ray regime and yields the resonant X-ray difference-frequency amplitude. In what follows, we work at the molecular level and, for isotropic samples, use the orientationally averaged single-molecule emission intensity as the observable.

### Quadratic Response and Difference-Frequency Amplitude

The derivation is carried out in the time domain, and frequency components are reported by using the standard Fourier convention. Energy conservation enters through the spectral Dirac delta δ­(ω_3_ – ω_1_ + ω_2_), which selects the component at the difference frequency for nearly monochromatic fields. In this two-level picture, the second-order response arises from virtual transitions that couple the dipole and quadrupole pathways through the same excited state. The process can be represented by a double-sided Feynman diagram in which one photon at ω_1_ is absorbed and one at ω_2_ is emitted, with radiation generated at ω_3_ = ω_1_ – ω_2_ as the system returns to the ground state. No real population of an intermediate state is involved, only coherence between the two levels, which is captured by the two-level propagator in (eq 7).

Second-order perturbation in the fields is retained while truncating the multipolar interaction at the dipole and quadrupole orders. Working in the interaction picture with respect to *H*_0_, the dipole operator μ̂^(0)^(*t*) evolves under *H*_0_, and to capture dipole–quadrupole interference, we include the first-order dressing of the dipole operator $H_{\mathrm{int}}^{\left(Q\right)}$ . At the molecular level, the second-order dipole moment reads

4 $$p^{\left(2\right)}\left(t\right)=\mathrm{Tr}\left[\rho^{\left(2\right)}\left(t\right){\mathrm{\mu ̂}}^{\left(0\right)}\left(t\right)\right]+\mathrm{Tr}\left[\rho^{\left(1\right)}\left(t\right){\mathrm{\mu ̂}}^{\left(1\right)}\left(t\right)\right]$$

with

5 $${\mathrm{\mu ̂}}^{\left(1\right)}\left(t\right)=\frac{i}{\hbar }\int_{-\infty }^{t}dt^{\prime }\left[H_{\mathrm{int}}^{\left(Q\right)}\left(t^{\prime }\right),{\mathrm{\mu ̂}}^{\left(0\right)}\left(t\right)\right]$$

6 $$H_{\mathrm{int}}^{\left(Q\right)}\left(t\right)=-\frac{1}{6}\mathrm{Q̂}\partial_{x}E\left(x,t\right)$$

which retains the μ–μ and μ–*Q* pathways and neglects *Q*–*Q* at this order.

Within a two-level description (|*a*⟩ → |*b*⟩) of transition frequency Ω_0_ and line width Γ, the molecular difference-frequency dipole at ω_3_ = ω_1_ – ω_2_ is

7 $$p^{\left(2\right)}\left(\omega_{3}\right)=\frac{A_{\mu \mu }+A_{\mu Q}}{\Omega_{0}-\left(\omega_{1}-\omega_{2}\right)-i\Gamma }E_{1}E_{2}^{*}$$

with

8 $$\begin{array}{l} A_{\mu \mu }=|\mu_{ab}{|}^{2},\\ A_{\mu Q}=\frac{i}{6}\left(k_{2}\mu_{ab}Q_{ba}+k_{1}Q_{ab}\mu_{ba}\right), \end{array}$$

where *k*_*j*_ = *ω*_*j*_/*c* and *μ*_*ab*_ = ⟨*a*|μ̂|*b*⟩, *Q*_*ab*_ = ⟨*a*|*Q̂*|*b*⟩. By Hermiticity $\mu_{ba}=\mu_{ab}^{*}$ and $Q_{ba}=Q_{ab}^{*}$ . The dipole–quadrupole pathway can carry an arbitrary phase, and when *μ*_*ab*_ *Q*_*ab*_ is real, it is in quadrature with the purely dipolar term. In the present plane-wave two-level model, the (ω_1_ + ω_2_)^2^ scaling of the observable correction is fixed by the electric dipole–quadrupole pathway through field gradients. Magnetic-dipole pathways enter at the same formal order but do not modify this scaling within the two-level description.

The same result can be viewed within the frequency-domain response formalism, where the molecular second-order polarization at the difference frequency is expressed as

9 $$p_{i}^{\left(2\right)}\left(\omega_{3}\right)=\varepsilon_{0}\beta_{ijk}\left(-\omega_{3};\omega_{1},\omega_{2}\right)E_{1j}E_{2k}^{*}$$

with *β*_*ijk*_(−ω_3_;ω_1_,ω_2_) being the quadratic response function.^15^ Comparison with eq 7 shows that, for parallel linear polarizations, the effective hyperpolarizability in the present two-level model reduces to

10 $$\beta_{\mathrm{eff}}\left(-\omega_{3};\omega_{1},\omega_{2}\right)=\frac{A_{\mu \mu }+A_{\mu Q}}{\varepsilon_{0}\left[\Omega_{0}-\left(\omega_{1}-\omega_{2}\right)-i\Gamma \right]}$$

with $A_{\mu \mu }$ and $A_{\mu Q}$ from eq 8. The orientational average acts on *β*_*ijk*_ *e*_1*j*_ *e*_2*k*_ and yields the isotropic intensity in eq 11, while the *k*-linear dipole–quadrupole pathway entering through $A_{\mu Q}$ produces the (ω_1_ + ω_2_) dependence leading to eq 14.

The single-molecule emission intensity at the difference frequency is obtained from the orientational average of the squared molecular response,

11 $$I\left(\omega_{3}\right)\propto \left\langle \right|p^{\left(2\right)}\left(\omega_{3}\right){|}^{2}\rangle_{\mathrm{orient}}$$

Expanding $|A_{\mu \mu }+A_{\mu Q}{|}^{2}$ at the molecular amplitude level yields a cross term 2Aμμ⁡Re(AμQ) , since $A_{\mu \mu }=|\mu_{ab}{|}^{2}$ is real. For isotropic samples measured in intensity with linear polarizations, this interference term cancels exactly upon orientational averaging, even though it is present at the amplitude level, so the orientationally averaged change is governed by the small positive quadratic term $|A_{\mu Q}{|}^{2}$ . This yields the quadratic *k* dependence used below. We focus on solvated samples because the ω_3_ field is strongly reabsorbed at a core edge; therefore, the relevant observable is the per-molecule conversion efficiency. The molecular form *I*(ω_3_) ∝ ⟨|*p* ^(2)^(ω_3_)|^2^⟩_orient_ is the same in gas and solution up to local-field factors *L*(ω) that multiply both pathways and cancel in δ*I*. Chiral gases can show a finite purely dipolar DFG or SFG under phase-sensitive geometries, but with linear polarizations and isotropic intensity averaging, this contribution vanishes and the measured change is governed by the surviving quadratic contribution originating from the *k*-linear beyond-dipole pathway at the amplitude level.

### Relative Quadrupole Correction

From eqs 7–8 and the intensity definition in eq 11, the change of the emitted intensity with respect to the purely dipolar pathway, obtained after the linear dipole–quadrupole interference term cancels upon orientational averaging, reads

12 $$\delta I≡\frac{{|A_{\mu \mu }+A_{\mu Q}{|}^{2}-\left|A_{\mu \mu }\right|}^{2}}{|A_{\mu \mu }{|}^{2}}={\left(\frac{k_{1}+k_{2}}{6}\right)}^{2}{\left(\frac{Q_{ab}}{\mu_{ab}}\right)}^{2}$$

For an isotropic ensemble, oscillator strengths follow the orientational average of the underlying tensor products. Using the irreducible electric quadrupole tensor,^8^ that is, the traceless rank-2 form removes the scalar trace and suppresses origin dependence, leaving only the genuine quadrupolar response. In atomic units, this gives

13 $$f^{\left(0\right)}=\frac{2}{3}\Omega_{0}\left|\mu_{ab}{|}^{2},,f^{\left(2\right)}=\frac{1}{20}\frac{\Omega_{0}^{3}}{c^{2}}\right|Q_{ab}{|}^{2}$$

In practice, the effective quadrupole parameter *Q*_*ab*_ entering this relation is inferred from the total isotropic beyond-dipole oscillator strength *f* ^(2)^ obtained from linear-response calculations in DIRAC. This avoids an artificial separation of individual multipolar contributions and provides a gauge-consistent parametrization of the beyond-dipole coupling within the effective two-level description. This leads to

14 $$\delta I=\frac{10}{27}\frac{{\left(\omega_{1}+\omega_{2}\right)}^{2}}{\Omega_{0}^{2}}\frac{\left|f^{\left(2\right)}\right|}{f^{\left(0\right)}}$$

In re-XDFG, the relevant small parameter is *k*_eff_ *r_c_* with *k*_eff_ ≡ *k*_1_ + *k*_2_ (not *k*_3_), because the dipole–quadrupole pathway arises from the gradient couplings ∂_*x*_ *E*_*j*_ = i*k*_*j*_ *E*_*j*_; thus eq 14 can be read as δ*I* ∝ (*k*_eff_r_c_)^2^|*f* ^(2)^|/*f* ^(0)^, where *r_c_* is the core length scale of the transition (e.g., the 1s extent).^6^ For the driving choice ω_1_ = 4Ω_0_ and ω_2_ = 3Ω_0_, one has (ω_1_ + ω_2_)^2^/Ω_0_^2^ = 49 and

15 $$\delta I=\frac{490}{27}\frac{\left|f^{\left(2\right)}\right|}{f^{\left(0\right)}}\approx 18.148\frac{\left|f^{\left(2\right)}\right|}{f^{\left(0\right)}}$$

In this formulation, the scaling in eqs 14–15 is fixed by the electric dipole–quadrupole pathway, while magnetic–dipole effects, if present, only renormalize the overall beyond–dipole magnitude within the two-level model. Accordingly, the use of an isotropic linear–response descriptor is quantitatively justified when the magnetic contribution is subdominant in the spectral window of interest. We use isotropic oscillator strengths defined as the rotational average over molecular orientations with the standard polarization sum, so eq 13 applies directly to the linearly polarized fields considered here. The beyond-dipole correction is quantified through the magnitude ratio |*f* ^(2)^/*f* ^(0)^|. The sign of *f* ^(2)^ is convention-dependent within a truncated multipolar description, but it is not required to evaluate δ*I* because the observable change depends on $|A_{\mu Q}{|}^{2}$ and is therefore positive and small, reflecting a quadratic contribution that originates from dipole–quadrupole interference at the amplitude level and survives only in the isotropically averaged intensity, as quantified in eqs 14 and 15.

## Computational Methods and Gauge Checks

We performed four-component linear-response DFT with the PBE0 hybrid functional. The uncontracted dyall.v3z basis was used for all atoms. Calculations employed closed-shell SCF and Gaussian nuclear charge distributions. In a single run, DIRAC prints the truncated multipole expansion and the full interaction, each in the generalized velocity and generalized length gauges. Unless noted otherwise, we used the truncated multipole values in the generalized velocity gauge for *f* ^(0)^ and *f* ^(2)^. Outputs from the generalized length gauge and from the full interaction were used as internal consistency checks. Transitions with very small *f* ^(0)^ in the velocity gauge were discarded to avoid numerical outliers.

As a reference, the CO bond length was set to 1.128 Å, with the molecular *z*-axis defined along the C–O bond. The *C*_2v_ double group symmetry was used.

On the strong dipole-allowed features used for the analysis, the generalized length gauge reproduces *f* ^(0)^ within numerical tolerance. The full interaction yields a total strength consistent with the truncated multipole result in the same spectral window. Reported *f* ^(2)^ values in the velocity gauge can be negative. Since the observable is the isotropically averaged single-molecule intensity and the dipole–quadrupole cross term vanishes for linear polarizations, only $|A_{\mu Q}{|}^{2}$ contributes. The net change is positive and small, as expected from the *k* ^2^ scaling. Analytical derivations and orientational-averaging identities are provided in the Supporting Information.

## Results and Discussion

We go beyond the electric-dipole approximation by evaluating linear, isotropic oscillator strengths with DIRAC.^16^ Within the truncated multipole expansion, the dipole contribution *f* ^(0)^ and the total isotropic beyond-dipole contribution *f* ^(2)^ are obtained in the generalized velocity gauge, with the generalized length gauge used for internal consistency checks. The full light–matter interaction, here meaning the minimal-coupling Hamiltonian without multipole truncation that retains the spatial dependence of the field as implemented in DIRAC and that we use as a gauge and origin check, is also evaluated to assess gauge and origin invariance and to quantify dipole-forbidden intensity, but it returns a total strength rather than a clean split into *f* ^(0)^ and *f* ^(2)^, so quoted *f* ^(0)^ and *f* ^(2)^ values are taken from the velocity gauge.

In this work, *f* ^(2)^ denotes the total isotropic beyond-dipole oscillator strength obtained from linear response and is used as the quantitative input to define the effective quadrupole parameter *Q*_*ab*_ entering the two-level scaling law. The ratio |*f* ^(2)^|/*f* ^(0)^ is therefore used as a diagnostic measure of nondipole coupling strength, while the frequency scaling of the observable correction is fixed by the electric dipole–quadrupole channel.

For CO at the O *K* edge and cysteine at the S *K* edge, length and velocity formulations are mutually consistent on dipole-allowed features, and both agree with the full interaction within numerical tolerance in the spectral windows considered. The beyond-dipole impact on the emitted difference-frequency intensity is quantified via the ratio |*f* ^(2)^|/*f* ^(0)^ as detailed below. We report two representative cases (a light and a mid-*Z K* edge) that typify the scaling. Extending the tables to other edges would not alter the conclusions: for the main dipole-allowed feature, the line-by-line spread of δ*I* is small and follows the same *R* trend, and the prefactor (ω_1_ + ω_2_)^2^/Ω_0_^2^ captures the *k*_eff_ *r*_c_ dependence. Matrix elements of the full light–matter interaction are available in DIRAC, and we use them as a gauge and origin check. In principle, one could construct the quadratic response β­(−ω_3_;ω_1_,ω_2_) with the full field operator. However, the full operator returns a total strength under isotropic averaging with no clean separation into dipole and beyond-dipole parts, so the parameter-free ratio |*f* ^(2)^|/*f* ^(0)^ cannot be formed directly. Our scaling therefore uses the generalized velocity-gauge decomposition, which provides *f* ^(0)^ and the total beyond-dipole strength *f* ^(2)^ in a consistent way for the spectral features considered. A full operator quadratic response evaluation would be valuable for future work but is beyond the scope of this study.

### Carbon Monoxide (CO)

We evaluate isotropic oscillator strengths at the O *K* edge of CO within linear-response theory. The truncated multipole expansion provides *f* ^(0)^ and the total isotropic beyond-dipole strength *f* ^(2)^ in the generalized velocity gauge, with the generalized length gauge used as an internal check and the full interaction used to assess gauge and origin invariance. We focus on the strongest features near the edge and list representative features in Table 1. To avoid numerically fragile lines, we discard transitions whose isotropic dipole strength *f* ^(0)^ in the velocity gauge is below 1.0 × 10^–8^. The table reports the transition energy in eV, the isotropic oscillator strengths *f* ^(0)^ and |*f* ^(2)^| from the truncated velocity gauge, and the corresponding transition moments μ_*ab*_ and *Q*_*ab*_ inferred via eq 13. The last column gives the estimated relative correction δ*I* to the emitted difference-frequency intensity from eq 14 for ω_1_ = 4Ω_0_ and ω_2_ = 3Ω_0_.

**Table 1:** Transition Moments and Estimated Relative Intensity Corrections for CO at the Oxygen *K* Edge (Ω_0_ ≈ 535 eV)^a^

Level	Energy (eV)	*f* ^(0)^	|*f* ^(2)^|	μ_*ab*_ (a.u.)	*Q*_*ab*_ (a.u.)	δ*I* (%)
3	527.1	6.15 × 10^–4^	4.60 × 10^–7^	0.006901	0.004876	1.357
6	528.5	1.46 × 10^–3^	1.07 × 10^–6^	0.010619	0.007407	1.330
7	531.2	7.20 × 10^–3^	5.21 × 10^–6^	0.023520	0.016217	1.313
10	537.0	2.67 × 10^–2^	1.93 × 10^–5^	0.045049	0.030710	1.312

a δ*I* computed for ω_1_ = 4Ω_0_, ω_2_ = 3Ω_0_. The reported *f* ^(2)^ values correspond to the total isotropic beyond-dipole oscillator strength obtained from linear response. The associated *Q*_*ab*_ values are effective parameters inferred from *f* ^(2)^ through eq 13 and should be interpreted within the effective two-level description. μ_*ab*_ and *Q*_*ab*_ are in atomic units (A.U.).

The main trend is a smooth, percent-level beyond-dipole correction across the dominant dipole-allowed features. For the strongest line, Level 10 at 537.0 eV in Table 1, we obtain *f* ^(0)^ = 2.67 × 10^–2^ and |*f* ^(2)^| = 1.93 × 10^–5^, which gives |*f* ^(2)^|/*f* ^(0)^ ≈ 7.2 × 10^–4^ and δ*I* ≈ 1.31%. Neighboring strong lines yield essentially the same ratio, 1.31–1.36%, indicating that the correction behaves as a broadband modulation governed primarily by the intrinsic *Q*/μ ratio of each transition together with the (ω/*c*)^2^ scaling, rather than by narrow spectral structure. The agreement among length, velocity, and full formulations on these features supports the effective gauge and origin insensitivity of the values used for our estimates.

Although CO is a linear molecule, it is not centrosymmetric because the carbon and oxygen atoms are inequivalent. It therefore possesses a small permanent dipole moment, and its second-order difference-frequency signal is finite already within the electric-dipole approximation. The reported δ*I* ≈ 1.31% represents the relative beyond-dipole enhancement with respect to this nonzero dipolar intensity. In contrast, for truly centrosymmetric species such as CO_2_, the dipolar contribution would vanish after isotropic averaging, and only the beyond-dipole pathway would remain.

### Cysteine

At the S *K* edge of cysteine, we extract isotropic oscillator strengths *f* ^(0)^ and the total isotropic beyond-dipole strength *f* ^(2)^ and use eqs 13–14 to estimate the beyond-dipole impact on the emitted difference-frequency intensity. Table 2 lists representative core-level transitions together with the corresponding transition moments and the resulting relative correction δ*I* for the driving choice ω_1_ = 4Ω_0_ and ω_2_ = 3Ω_0_. For the two strongest features reported, the magnitude ratio is |*f* ^(2)^|/*f* ^(0)^ ≈ 3.05 × 10^–3^, which yields δ*I* ≈ 5.53%. The close agreement between these lines indicates a smooth modulation governed primarily by the intrinsic *Q*/μ ratio over the window considered and is consistent with the scaling in eq 13.

**Table 2:** Transition Moments and Estimated Relative Corrections for Cysteine at the Sulfur *K* Edge (Ω_0_ ≈ 2427 eV)^a^

Level	Energy (eV)	*f* ^(0)^	|*f* ^(2)^|	μ_*ab*_ (a.u.)	*Q*_*ab*_ (a.u.)	δ*I* (%)
4	2426.78	6.83 × 10^–4^	2.08 × 10^–6^	0.003389	0.001049	5.53
8	2427.54	4.30 × 10^–4^	1.31 × 10^–6^	0.002689	0.000832	5.53

a δ*I* computed for ω_1_ = 4Ω_0_, ω_2_ = 3Ω_0_. The reported *f* ^(2)^ values correspond to the total isotropic beyond-dipole oscillator strength obtained from linear response. The associated *Q*_*ab*_ values are effective parameters inferred from *f* ^(2)^ through eq 13 and should be interpreted within the effective two-level description. μ_*ab*_ and *Q*_*ab*_ are in atomic units (A.U.)

The quadrupolar correction provides additional chemical insight by revealing how the nonlinear X-ray response becomes sensitive to the spatial extent and local symmetry of the core orbital involved in the transition. While the dipolar approximation reflects mainly the total oscillator strength, the quadrupolar term emphasizes the anisotropy of the core region and thus carries element- and site-specific information. The percent-level increase reported here quantifies this additional sensitivity. Comparison with the full light–matter interaction in DIRAC confirms that the beyond-dipole correction captured by the E2 + M1 term reproduces the total response within numerical accuracy in the relevant spectral window, indicating that higher multipoles are not required at the *K* edges studied.

### Nondipole Difference-Frequency Corrections

To condense the spectral information into a single parameter per edge, we average the isotropic oscillator strengths over a narrow window centered at the dominant dipole feature. Using weights proportional to *f* ^(0)^, we define the weighted averages

$$\overset{¯}{f^{\left(0\right)}}=\frac{\underset{j}{\sum }(f_{j}^{\left(0\right)}{)}^{2}}{\underset{j}{\sum }f_{j}^{\left(0\right)}},,\overset{―}{\left|f^{\left(2\right)}\right|}=\frac{\underset{j}{\sum }\left|f_{j}^{\left(2\right)}\right|f_{j}^{\left(0\right)}}{\underset{j}{\sum }f_{j}^{\left(0\right)}}$$

and the working ratio

$$R≡\frac{\overset{―}{\left|f^{\left(2\right)}\right|}}{\overset{¯}{f^{\left(0\right)}}}$$

For CO at the O *K* edge, we obtain $R_{\mathrm{CO}}\approx 7.2\times 10^{-4}$ , while for cysteine at the S *K* edge, we find $R_{\mathrm{Cys}}\approx 3.05\times 10^{-3}$ , consistent with Tables 1 and 2 and with eq 13). Inserting R into eq 14 gives the relative nondipole correction to the emitted difference-frequency intensity at ω_3_ = ω_1_ – ω_2_

$$\delta I\left(r\right)=\frac{10}{27}{\left(2r-1\right)}^{2}R$$

which we report as percentages 100 × δ*I*(*r*). The scaling in eq 14 implies a monotonic increase of the nondipole correction with the core energy. The quantities *f* ^(2)^ ∝ Ω_0_^3^|*Q*|^2^/*c* ^2^ and *k*_eff_ = (ω_1_ + ω_2_)/*c* both grow with the edge energy, so R and hence δ*I* tend to be larger at heavier *K* edges and at deep *L* edges than at lighter ones. Consequently, the CO C *K* edge is expected to exhibit a smaller correction than the *K* edge under otherwise identical driving. Line-by-line values can be read directly from $f_{j}^{\left(0\right)}$ and $\left|f_{j}^{\left(2\right)}\right|$ . For strong dipole-allowed features, the spread in δ*I*_*j*_ is modest, which justifies the compact weighted average R . Dipole-forbidden features can appear in the full interaction through beyond-dipole couplings. In those cases, the relative metric δ*I* defined against the dipolar pathway is not applicable, and absolute single-molecule intensities from the full interaction should be considered for experimental targeting.

In phase-sensitive heterodyne detection with a coherent local oscillator *E*_LO_, the measured signal is Shet∝Re[ELO*p(2)(ω3)] . Using eq 7, this yields a relative correction that is linear in the dipole-quadrupole pathway,

ΔShetShet(0)≃Re{AμQAμμeiϕLO}

which can be tuned positive or negative by the local-oscillator phase ϕ_LO_. From eqs 8 and 13, the magnitude is bounded by

$$\left|\frac{\Delta S_{het}}{S_{het}^{\left(0\right)}}\right|≲\frac{k_{1}+k_{2}}{6}\left|\frac{Q_{ab}}{\mu_{ab}}\right|∼\sqrt{\frac{10}{27}}\frac{\omega_{1}+\omega_{2}}{\Omega_{0}}\sqrt{\frac{\left|f^{\left(2\right)}\right|}{f^{\left(0\right)}}}$$

Thus, unlike the homodyne case, where the cross term cancels after isotropic averaging, a heterodyne geometry that preserves interference can reveal chemically specific phase contrasts. A complete treatment for isotropic liquids requires a defined local-oscillator polarization and possibly partial alignment and is left for future work.

Figure 1 shows 100 × δ*I*(*r*) over 2.4 ≤ *r* ≤ 4.5. The curves follow eq 14 using the averaged R values defined above. The growth with *r* reflects the (2*r* – 1)^2^ dependence of the *k*-linear dipole–quadrupole pathway, and the vertical offset between edges follows from the larger R at higher core energies. Gauge checks with the generalized length and velocity formulations and with the full interaction show negligible differences in the features entering the averages, supporting the robustness of R for both systems. After isotropic averaging with linear polarizations, the interference term cancels, and the remaining change is the small positive quadratic contribution shown in Figure 1.

**Figure 1:**
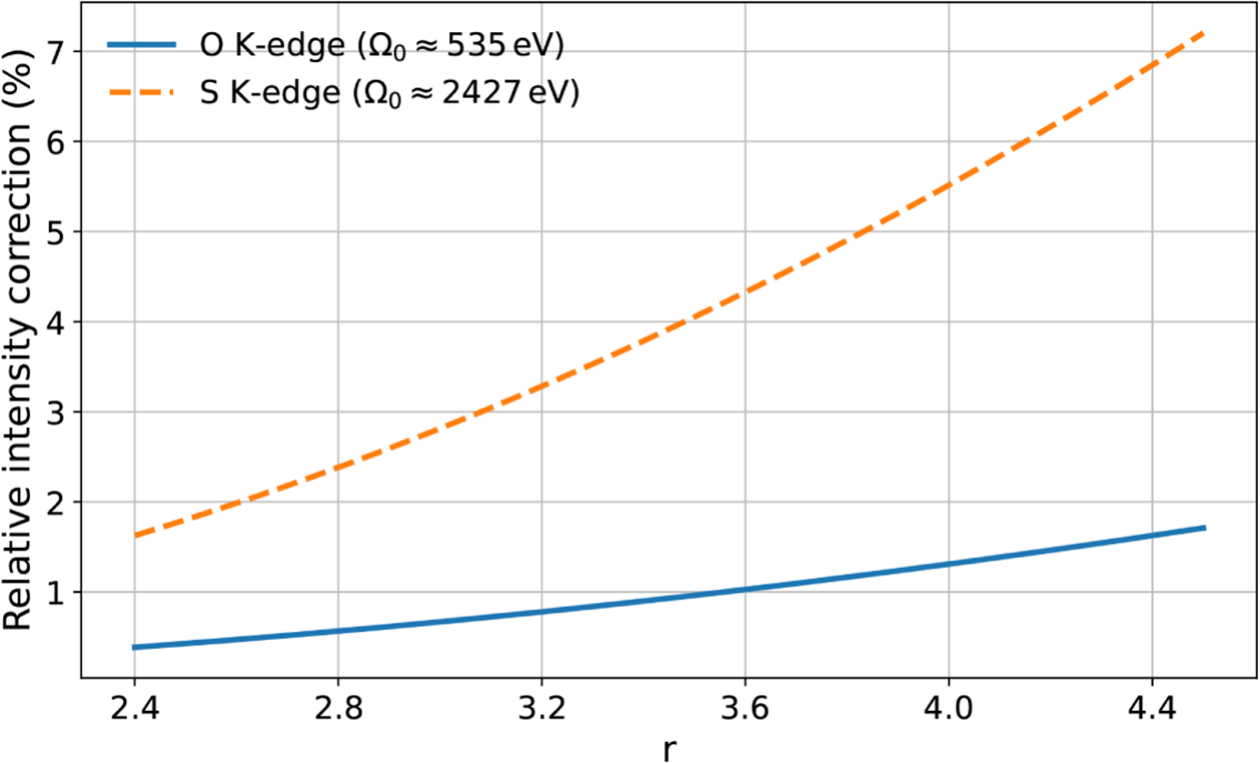
Relative nondipole correction to the difference-frequency intensity, 100 × *δI*(*r*) _0_, obtained from eq 14. The shown lines use weighted averages of the isotropic oscillator strengths around the main dipole feature to define $R=\overset{¯}{\left|f^{\left(2\right)}\right|}/\overset{¯}{f^{\left(0\right)}}$ (CO, O *K* edge: $R\approx 7.2\times 10^{-4}$ ; cysteine, S *K* edge: $R\approx 3.05\times 10^{-3}$ ). The growth with *r* follows the (2*r* – 1)^2^ factor from the dipole–quadrupole pathway, and the larger correction at the S *K* edge reflects the intrinsic increase of the quadrupolar response with core energy. Magnitudes are shown. The sign of *f* ^(2)^ is convention-dependent within the truncated multipolar description.

For guiding measurements, the key point is the sign. For isotropic samples measured in intensity with linear polarizations, the cross term 2Aμμ⁡Re(AμQ) averages to zero by symmetry, so the orientationally averaged change is dominated by $|A_{\mu Q}{|}^{2}$ and yields a modest increase by the percentages in Figure 1. In contrast, oriented samples or polarization geometries that preserve interference can produce either an increase or a decrease, depending on the relative phase set by the setup. Resolving the sign in those cases requires phase-sensitive detection or a fully gauge-consistent calculation that fixes the relative phase for the chosen geometry.

## Conclusion

We introduced a compact two-level framework for second-order X-ray response beyond the electric dipole, including the quadrupolar correction and its interference with the dipolar pathway. The approach links the magnitude of the nondipole effect to weighted linear oscillator strengths, enabling quick estimates from standard linear-response data. Under isotropic conditions with linear polarizations and for the two-color choice used here, the predicted change in the emitted difference-frequency intensity is about 1.3% at the O *K* edge of CO and about 5.5% at the S *K* edge of cysteine, identifying regimes where a dipole-only analysis becomes insufficient.

The model targets molecules in the gas phase or solution with isotropic orientational averaging of the emitted intensity from single molecules. It does not address crystalline solids, where band structure, crystal symmetry, and local-field effects govern the tensor response and must be treated with Bloch states and the crystal χ^(2)^. Higher multipoles and nearby continua are neglected, and a single resonant denominator is used, so the dispersive structure and pulse bandwidth can reshape line shapes in time-domain measurements.

Although we focused on difference-frequency generation, the same molecular treatment applies to sum-frequency generation (SFG) by permuting the input frequencies in the two-level response *p* ^(2)^ and evaluating it at ω_3_ = ω_1_ + ω_2_. When SFG is tuned to the same core transition, (ω_1_ + ω_2_)/Ω_0_ ≃ 1, so the dipole–quadrupole prefactor proportional to (ω_1_ + ω_2_)^2^/Ω_0_^2^ is of order unity rather than 49 as in the DFG case. Consequently, the predicted nondipole correction is smaller by roughly 1–2 orders of magnitude, about 0.03% for CO and 0.11% for cysteine with the present parameters. The sign of the correction remains geometry- and phase-sensitive in both DFG and SFG, so intensity predictions should be interpreted as magnitudes unless phase control or a fully gauge-consistent phase assignment is available.

In summary, the framework provides a practical tool for experimental planning. It allows one to estimate the magnitude of beyond-dipole effects from linear spectra and to identify when a dipole-only description is no longer sufficient, while the sign of the response remains geometry- and phase-dependent. For isotropic samples measured in intensity with linear polarizations, the cross term cancels upon isotropic orientational averaging; therefore, the observable change is the small positive quadratic correction quantified above. Viewed as a design rule, the present scaling connects microscopic beyond-dipole matrix elements to percent-level efficiency changes that remain visible even when the macroscopic ω_3_ field is reabsorbed. Although derived for re-XDFG, the same two-level structure extends to SFG with a smaller nondipole prefactor, providing a unified framework for second-order X-ray mixing beyond the electric dipole.

## Supplementary Material



## Notes

Additional theoretical derivations, orientational averaging identities, and details of the treatment of magnetic–dipole contributions (PDF)
