# Supplementary material for: Two-Level Theory of Second-Order Nonlinear X‑ray Response beyond the Electric-Dipole Approximation
Source: J Phys Chem A. 2026 Jan 13;130(4):823–9. doi: 10.1021/acs.jpca.5c07630 (PMC12862796; doi:10.1021/acs.jpca.5c07630)
Supplement: Supplementary file 1 [file jp5c07630_si_001.pdf]

Supporting Information for Two-Level Theory of Second-Order Nonlinear X–Ray Response Beyond the Electric–Dipole Approximation

Abhinay V. Mohan[1,2] and Carles Serrat[1]

[1]Department of Physics, Universitat Politècnica de Catalunya, Ronda de Sant Nebridi 22, 08222 Terrassa, Spain
[2]Aix–Marseille Université, Faculté de Physique, 13013 Marseille, France

Supporting Information

S1. Conventions, geometry, and working formulas

We use bold symbols for vectors and rank–2 tensors and hats for operators. Matrix elements inherit boldface. Thus $\hat{\boldsymbol{\mu}}$ is the electric–dipole operator, $\hat{\mathbf{Q}}$ the irreducible (traceless) electric quadrupole operator, and $\hat{\mathbf{m}}$ the magnetic–dipole operator. We write $\boldsymbol{\mu}_{ab} \equiv \langle a|\hat{\boldsymbol{\mu}}|b\rangle$, $\mathbf{Q}_{ab} \equiv \langle a|\hat{\mathbf{Q}}|b\rangle$, and $\mathbf{m}_{ab} \equiv \langle a|\hat{\mathbf{m}}|b\rangle$. Unless noted we use atomic units and c denotes the speed of light.

We consider two monochromatic plane waves propagating along x with linear polarizations,

$$\mathbf{E}_j(x,t) = \mathbf{e}_j E_j\, e^{i(k_j x - \omega_j t)} + \text{c.c.}, \qquad k_j = \omega_j/c, \qquad j = 1, 2,$$

with Fourier convention $e^{-i\omega t}$ for the local fields at the molecular center $x = 0$. For a plane wave,

$$\mathbf{B}_j(x,t) = \frac{1}{c}\,\hat{\mathbf{x}} \times \mathbf{E}_j(x,t),$$

so magnetic fields are suppressed by $1/c$ relative to electric fields, while electric–quadrupole couplings are suppressed by the field gradient $\partial_x \mathbf{E}_j = ik_j \mathbf{E}_j$.

The observable used throughout this work is the orientationally averaged single–molecule emission intensity at the difference frequency,

$$I(\omega_3) \propto \left\langle \left|p^{(2)}(\omega_3)\right|^2 \right\rangle_{\text{orient}}, \qquad \omega_3 = \omega_1 - \omega_2,$$

for an isotropic ensemble and linear polarizations.

Numerical constants used in post–processing:

$$1 \text{ Hartree} = 27.211386245988 \text{ eV}, \qquad c = 137.035999084.$$

S2. Multipolar light–matter Hamiltonian to order kr_c

In the semi–classical multipolar gauge, truncated consistently to order kr_c, the interaction Hamiltonian reads

$$\hat{H}_\text{int}(t) = -\,\hat{\boldsymbol{\mu}} \cdot \mathbf{E}(x,t) - \frac{1}{6}\,\hat{\mathbf{Q}} : \nabla\mathbf{E}(x,t) - \,\hat{\mathbf{m}} \cdot \mathbf{B}(x,t) + \ldots \tag{S1}$$

where the electric quadrupole operator is irreducible (traceless). For propagation along x we keep the spatial phase so that $\partial_x \mathbf{E}_j = ik_j \mathbf{E}_j$, and we evaluate the local response at the molecular center $x = 0$. The target frequency is $\omega_3 = \omega_1 - \omega_2$. We denote the detuning by $\Lambda = \Omega_0 - (\omega_1 - \omega_2)$ and the common Lorentzian factor by $[\Lambda - i\Gamma]^{-1}$.

S3. Two–level second–order amplitude including E2 and M1

For a two–level transition $|a\rangle \to |b\rangle$ of frequency Ω_0 and width Γ, the molecular second–order dipole at ω_3 can be written as

$$p^{(2)}(\omega_3) = \frac{\mathcal{A}_{\mu\mu} + \mathcal{A}_{\mu Q} + \mathcal{A}_{\mu M}}{\Omega_0 - (\omega_1 - \omega_2) - i\Gamma}\, E_1 E_2^*, \tag{S2}$$

where the three compact amplitudes correspond to the dipole–dipole pathway and the two beyond–dipole (BD) pathways that enter at order kr_c:

$$\mathcal{A}_{\mu\mu} = (\mathbf{e}_1\cdot\boldsymbol{\mu}_{ab})(\mathbf{e}_2\cdot\boldsymbol{\mu}_{ba}), \tag{S3}$$

$$\mathcal{A}_{\mu Q} = \frac{i}{6}\Big[k_2\,(\mathbf{e}_2\cdot\boldsymbol{\mu}_{ab})\,(\mathbf{e}_1\mathbf{e}_1:\mathbf{Q}_{ba}) + k_1\,(\mathbf{e}_1\cdot\boldsymbol{\mu}_{ba})\,(\mathbf{e}_2\mathbf{e}_2:\mathbf{Q}_{ab})\Big], \tag{S4}$$

$$\mathcal{A}_{\mu M} = \frac{1}{c}\Big[(\mathbf{e}_2\cdot\boldsymbol{\mu}_{ab})\,\big((\hat{\mathbf{x}}\times\mathbf{e}_1)\cdot\mathbf{m}_{ba}\big) + (\mathbf{e}_1\cdot\boldsymbol{\mu}_{ba})\,\big((\hat{\mathbf{x}}\times\mathbf{e}_2)\cdot\mathbf{m}_{ab}\big)\Big]. \tag{S5}$$

By Hermiticity $\boldsymbol{\mu}_{ba} = \boldsymbol{\mu}^*_{ab}$, $\mathbf{Q}_{ba} = \mathbf{Q}^*_{ab}$, and $\mathbf{m}_{ba} = \mathbf{m}^*_{ab}$. The key structural point is that $\mathcal{A}_{\mu Q}$ is proportional to k_1 and k_2, while $\mathcal{A}_{\mu M}$ is proportional to $1/c$ but carries no additional factor of k_j from spatial gradients. This difference controls the frequency scaling of the correction.

For compactness we define the total beyond–dipole amplitude

$$\mathcal{A}_{\rm BD} \equiv \mathcal{A}_{\mu Q} + \mathcal{A}_{\mu M}.$$

S4. Orientational average and the general form of the scaling

The intensity observable is

$$I(\omega_3) \propto \frac{|E_1|^2|E_2|^2}{[\Omega_0 - (\omega_1-\omega_2)]^2 + \Gamma^2}\Big\langle\, |\mathcal{A}_{\mu\mu} + \mathcal{A}_{\rm BD}|^2 \,\Big\rangle_{\rm orient}. \tag{S6}$$

Expanding the modulus squared yields

$$\Big\langle\, |\mathcal{A}_{\mu\mu} + \mathcal{A}_{\rm BD}|^2 \,\Big\rangle_{\rm orient} = \Big\langle\, |\mathcal{A}_{\mu\mu}|^2 \,\Big\rangle_{\rm orient} + \Big\langle\, |\mathcal{A}_{\rm BD}|^2 \,\Big\rangle_{\rm orient} + 2\Big\langle\, {\rm Re}\big(\mathcal{A}^*_{\mu\mu}\mathcal{A}_{\rm BD}\big) \,\Big\rangle_{\rm orient}. \tag{S7}$$

For isotropic ensembles measured in intensity with linear polarizations, all dipole–beyond–dipole interference terms, while present at the amplitude level, vanish by symmetry upon orientational averaging, so

$$\Big\langle\, |\mathcal{A}_{\mu\mu} + \mathcal{A}_{\rm BD}|^2 \,\Big\rangle_{\rm orient} = \Big\langle\, |\mathcal{A}_{\mu\mu}|^2 \,\Big\rangle_{\rm orient} + \Big\langle\, |\mathcal{A}_{\rm BD}|^2 \,\Big\rangle_{\rm orient}. \tag{S8}$$

We therefore define the observable relative correction as

$$\delta I \equiv \frac{\Big\langle\, |\mathcal{A}_{\rm BD}|^2 \,\Big\rangle_{\rm orient}}{\Big\langle\, |\mathcal{A}_{\mu\mu}|^2 \,\Big\rangle_{\rm orient}}. \tag{S9}$$

Using $\mathcal{A}_{\rm BD} = \mathcal{A}_{\mu Q} + \mathcal{A}_{\mu M}$, the correction decomposes into

$$\delta I = \delta I_{\mu Q} + \delta I_{\mu M} + \delta I_{\mu Q\mu M}, \tag{S10}$$

with

$\delta I_{\mu Q} \propto (k_1+k_2)^2$ (electric–quadrupole channel), $\quad \delta I_{\mu M} \propto \dfrac{1}{c^2}$ (magnetic–dipole channel),

and $\delta I_{\mu Q \mu M}$ the E2–M1 cross term within $|\mathcal{A}_{\rm BD}|^2$. For the isotropic, linearly polarized intensity observable considered here, the BD cross term is symmetry–suppressed, so the correction is well approximated by the sum of the two positive quadratic contributions,

$$\delta I \simeq \delta I_{\mu Q} + \delta I_{\mu M}. \tag{S11}$$

The electric dipole–quadrupole term $\delta I_{\mu Q}$ yields the analytic prefactor $(\omega_1+\omega_2)^2/\Omega_0^2$ derived in the main text, while the magnetic dipole contribution originates from $\mathbf{B}_j = (1/c)\hat{\mathbf{x}} \times \mathbf{E}_j$ rather than from spatial gradients of $\mathbf{E}_j$ and therefore does not introduce an independent $(\omega_1 + \omega_2)^2$ scaling within the two–level description of the isotropically averaged intensity. Therefore, within the two–level description and for the isotropic, linearly polarized intensity observable considered here, the electric dipole–quadrupole channel fixes the analytic $(\omega_1+\omega_2)^2/\Omega_0^2$ scaling, while magnetic–dipole contributions can only renormalize the overall magnitude of the correction.

Connection to isotropic oscillator strengths and DIRAC.

In linear response, DIRAC reports the isotropic beyond–dipole oscillator strength split into three pieces, labeled $Q-Q$, $M-Q$, and $M-M$, which add to the printed $f^{(2)}$ in the generalized velocity (or length) gauge. In this work we use

$$f^{(2)}_{\rm tot} = f^{(2)}_{Q\text{–}Q} + f^{(2)}_{M\text{–}Q} + f^{(2)}_{M\text{–}M}$$

as a diagnostic measure of nondipole coupling strength at a given edge. The two–level intensity correction δI for the isotropic, linearly polarized observable is controlled by the quadratic BD contribution $\langle |\mathcal{A}_{\rm BD}|^2 \rangle_{\rm orient}$. The electric–quadrupole part reproduces the analytic $(\omega_1+\omega_2)^2$ scaling of the isotropically averaged intensity, while the magnetic–dipole part contributes an additional positive quadratic term that does not introduce an independent scaling law within the two–level description.

S5. CO at the O K edge – numerical summary

Line–by–line isotropic oscillator strengths for CO at the O K edge are reported in Table 1 of the main text. To avoid duplication, we only summarize conventions and the aggregate used in Fig. 1. Values are taken from the truncated multipole expansion in the generalized velocity gauge, with generalized length and full interaction used as internal checks. Lines with very small velocity–gauge $f^{(0)}$ are discarded using a conservative threshold $f^{(0)} < 1.0 \times 10^{-8}$.

Over a narrow window around the dominant dipole feature, we define the weighted averages

$$\overline{f^{(0)}} = \frac{\sum_j \big(f_j^{(0)}\big)^2}{\sum_j f_j^{(0)}}, \qquad \overline{|f^{(2)}|} = \frac{\sum_j |f_j^{(2)}|\, f_j^{(0)}}{\sum_j f_j^{(0)}},$$

and the ratio $\mathcal{R} = \overline{|f^{(2)}|}/\overline{f^{(0)}}$. For CO we obtain $\mathcal{R}_{\rm CO} \approx 7.21 \times 10^{-4}$, which enters

$$\delta I(r) = \frac{10}{27}\,(2r-1)^2\,\mathcal{R}_{\rm CO}, \qquad r = \omega_1/\Omega_0, \;\; \omega_2 = \omega_1 - \Omega_0.$$

S6. Cysteine at the S K edge – numerical summary

Line–by–line isotropic oscillator strengths for cysteine at the S K edge are reported in Table 2 of the main text, with the same conventions as in Sec. S1. Using the same weighting in a narrow window around the main dipole feature we obtain

$$\mathcal{R}_{\text{Cys}} \approx 3.05 \times 10^{-3},$$

which enters the scaling

$$\delta I(r) = \frac{10}{27}\,(2r-1)^2\,\mathcal{R}_{\text{Cys}}.$$

S7. How to reproduce the numbers from a DIRAC output

1. Run linear response and request isotropic oscillator strengths. In one job DIRAC reports both the truncated multipole expansion and the full interaction, each in the generalized velocity and generalized length gauges. Use the truncated multipole values in the generalized velocity gauge for $f^{(0)}$ and for the total beyond–dipole oscillator strength $f^{(2)}$, which includes electric–quadrupole and magnetic–dipole contributions. Use the generalized length and full interaction outputs as internal checks.

2. Discard lines with $f^{(0)}$ below a small threshold in the velocity gauge to avoid numerical outliers. A value like 1.0×10^{-8} works for CO.

3. In a narrow window around the main dipole feature compute

$$\overline{f^{(0)}} = \frac{\sum_j \left(f_j^{(0)}\right)^2}{\sum_j f_j^{(0)}}, \qquad \overline{|f^{(2)}|} = \frac{\sum_j |f_j^{(2)}|\, f_j^{(0)}}{\sum_j f_j^{(0)}}$$

   and the ratio $\mathcal{R} = \overline{|f^{(2)}|}/\overline{f^{(0)}}$.

4. Insert $\mathcal{R}$ into

$$\delta I(r) = \frac{10}{27}\,(2r-1)^2\,\mathcal{R}, \qquad r = \omega_1/\Omega_0, \quad \omega_2 = \omega_1 - \Omega_0$$

   and report $100 \times \delta I$ as percent.

S8. Orientational identities used

For a unit vector $\mathbf{u}$,

$$\langle u_i u_j \rangle_{\text{orient}} = \frac{1}{3}\delta_{ij}, \qquad \langle u_i u_j u_k u_l \rangle_{\text{orient}} = \frac{1}{15}\left(\delta_{ij}\delta_{kl} + \delta_{ik}\delta_{jl} + \delta_{il}\delta_{jk}\right)$$

For a traceless symmetric rank 2 tensor Q_{ij},

$$\langle Q_{ij} \rangle_{\text{orient}} = 0, \qquad \langle Q_{ij} Q_{kl} \rangle_{\text{orient}} = \frac{1}{5}\,Q^2\,\Pi_{ijkl}, \quad \Pi_{ijkl} = \frac{1}{2}\left(\delta_{ik}\delta_{jl} + \delta_{il}\delta_{jk}\right) - \frac{1}{3}\delta_{ij}\delta_{kl}$$

These imply the vanishing of the dipole–quadrupole cross term under isotropic averaging with linear polarizations.

S9. Extension to sum frequency generation

The same response applies to SFG by evaluating $p^{(2)}$ at $\omega_3 = \omega_1 + \omega_2$ and permuting the frequencies in the amplitudes. When SFG is tuned to the same core transition $(\omega_1{+}\omega_2)/\Omega_0 \simeq 1$, the k–linear dipole–quadrupole prefactor proportional to $(\omega_1 + \omega_2)^2/\Omega_0^2$ is of order unity rather than 49 as in the re–XDFG choice $\omega_1 = 4\Omega_0$ and $\omega_2 = 3\Omega_0$. The predicted nondipole change in the isotropically averaged intensity is then smaller by roughly one to two orders of magnitude for the systems considered. The analytical frequency scaling derived in this work is therefore exact for the electric–dipole–electric–quadrupole channel and remains valid as a parametrization of the total beyond–dipole correction when magnetic–dipole contributions are included through $f^{(2)}$.
